# Impact of Dialysis Requirement in Community-acquired Pneumonia Hospitalizations

**DOI:** 10.7759/cureus.3164

**Published:** 2018-08-20

**Authors:** Uvesh Mansuri, Achint A Patel, Mihir Dave, Kinsuk Chauhan, Aakashi S Shah, Ramyasree Banala, David Ali, Saad Kamal, Pooja Verma, Shamim Ahmed, Prakash Maiyani, Ambarish C Pathak, Shajoti Rahman, Sejal Savani, Surta Pandya, Girish Nadkarni

**Affiliations:** 1 Internal Medicine, Medstar Union Memorial Hospital, Baltimore, USA; 2 Nephrology, The Icahn School of Medicine at Mount Sinai, New York, USA; 3 Nephrology, Icahn School of Medicine at Mount Sinai, New York, USA; 4 Cardiology, Icahn School of Medicine at Mount Sinai, New Rochelle, USA; 5 Public Health, Rutgers School of Public Health, Piscataway , USA; 6 Miscellaneous, Icahn School of Medicine at Mount Sinai, New York, USA; 7 Internal Medicine, Kings Brook Jewish Medical Center, New York, USA; 8 Miscellaneous, St. George’s University School of Medicine, St. George, GRD; 9 Internal Medicine, Gold Coast University Hospital, Southport, AUS; 10 Public Health, New York Medical, New York, USA; 11 SUNY Downstate College of Medicine, New York, USA; 12 New York University, New York, USA; 13 Wayne State University, Detroit, USA

**Keywords:** community acquired pneumonia, acute kidney injury

## Abstract

Background

Community-acquired pneumonia (CAP) is a common cause of hospitalization. While there are single-center studies on acute kidney injury requiring dialysis (AKI-D) and CAP, data on national trends and outcomes regarding AKI-D in CAP hospitalizations is lacking.

Methods

We utilized the Nationwide Inpatient Sample to analyze trends overall and within subgroups. We also utilized multivariate regression to adjust for potential confounders of annual trends and to generate adjusted odds ratios (aOR) for predictors and outcomes, including mortality and adverse discharge.

Results

There were 11,500,456 pneumonia hospitalizations between 2002 and 2013, of which 3675 (0.3%) were complicated by AKI-D. The AKI-D rate increased from 2.7/1000 hospitalizations in 2002 to 4.3/1000 hospitalizations in 2013. The rate of increase was higher in males and African Americans. Although temporal changes in demographics and comorbidities explained a substantial proportion, they could not explain the entire trend. The predictor with the highest odds of AKI-D required mechanical ventilation during hospitalization (aOR 12.47; 95% CI 11.66-13.34). Other significant predictors included sepsis (aOR 4.37; 95% CI 4.09-4.66), heart failure (aOR 2.40; 95% CI 2.25-2.55), and chronic kidney disease (CKD) (aOR 2.00; 95% CI 1.86-2.16). AKI-D was associated with increased in-hospital mortality (aOR 3.08; 95% CI 2.88-3.30) and adverse discharge (aOR 2.09; 95% CI 1.92-2.26). Although adjusted mortality decreased per year, attributable mortality remained stable.

Conclusion

Pneumonia hospitalizations complicated by AKI-D have increased with a differential increase by demographic groups. AKI-D is associated with significant morbidity and mortality. In the absence of effective AKI-D therapies, the focus should be on early risk stratification and prevention to avoid this devastating complication.

## Introduction

Community-acquired pneumonia (CAP) is one of the most common reasons for hospitalization in the United States; estimates ranging from 64-164 cases/10,000 adults [1-2]. This places a large burden on the health care system. Although most adults recover, it is a leading cause of mortality in adults [3].

Acute kidney injury (AKI) is a frequent complication in hospitalized patients, with an incidence of approximately 5%-7% of all hospitalized adults and nearly 20% of patients hospitalized with sepsis [4-5]. AKI is not only associated with morbidity and mortality but also with significant additional expenditures to the healthcare system [4].

Pneumonia is one of the most common causes of sepsis, with severe sepsis and septic shock developing in approximately 48% and 5%, respectively [6]. The epidemiology of AKI in CAP is understudied, with a majority of studies being single-center and cross-sectional [7-8]. Once AKI progresses to the most severe form of AKI, that requiring dialysis (AKI-D), the prognosis greatly worsens with mortality rates approaching 50% [9]. However, the impact of AKI-D on CAP hospitalizations has not been studied at a national level and, thus, we sought to explore the national epidemiology of AKI-D using a nationally representative database. The goals of this analysis were fourfold: (1) to assess secular temporal trends in AKI-D complicating CAP hospitalizations, overall and in demographic subgroups; (2) to explore reasons for changes in trends; (3) to elucidate acute/chronic conditions independently associated with AKI-D in CAP; and (4) to quantify the independent impact of AKI-D on outcomes, including mortality, morbidity, length of stay (LoS) and hospitalization cost.

## Materials and methods

Data sources

We extracted data from the Nationwide Inpatient Sample (NIS) of the Healthcare Cost and Utilization Project (HCUP) of the Agency for Healthcare Research and Quality (AHRQ) for years 2002–2013 [10]. This is the largest publicly available all-payer (including private insurance and uninsured) inpatient care database in the United States. It contains discharge-level data from 44 states that participate in the Healthcare Cost and Utilization Project, representing over 96% of the U.S. population. The NIS includes data from nearly 8-million hospital stays yearly from about 1,000 hospitals designed to approximate a 20% stratified sample of all hospitals in the United States. Discharge weights are provided for each patient discharge record and were used to obtain national estimates with high fidelity. As this was an administrative database, it does not contain granular data, such as laboratory results or timing of dialysis initiation.

Definition of CAP and AKI-D

We used the NIS database from 2002-2013 to identify adults hospitalized with CAP by using the AHRQ Clinical Classification Software (CCS), which groups International Classification of Diseases-9 Clinical Modification (ICD-9-CM) codes into mutually exclusive groups. This was chosen, as a combination of ICD-9-CM codes has better specificity and sensitivity for identifying CAP compared to individual codes [11]. The CCS category for pneumonia (122) includes all ICD-9-CM codes for pneumonia except for pneumonia caused by tuberculosis, sexually transmitted diseases, or influenza. We defined AKI using ICD-9-CM code 584.xx and dialysis by the procedure code of 39.95 or the diagnoses code of v45.11, v56.0, or v56.1. We excluded hospitalizations with procedure codes for arterio-venous access creation/revision since they were likely for the initiation of maintenance hemodialysis [4]. We excluded hospitalizations with dialysis codes but no AKI code, assuming that patients were receiving dialysis for end-stage renal disease (ESRD). This approach has been used previously and has high sensitivity and specificity [5]. Since ICD-9-CM codes for identifying AKI have been demonstrated to only have a sensitivity of 17%, we chose to focus on AKI-D instead of AKI [12].

Definition of outcomes

The outcomes of interest included in-hospital mortality, adverse discharge disposition, LoS, and hospitalization cost in US Dollars (USD). Discharge disposition was grouped into: (1) home or short-term facility versus (2) adverse discharge (skilled nursing facility, intermediate care, hospice home, hospice medical facility, long-term care hospital, or certified nursing facility).

Covariates

We extracted demographics (age, sex, and race), comorbidities (acute/chronic conditions, such as diabetes mellitus, chronic kidney disease, hypertension, sepsis, human immunodeficiency virus (HIV) status, liver disease, acute myocardial infarction, and heart failure) and procedures, including mechanical ventilation and estimated mortality risk using the validated All Patient Refined Diagnosis Related Group (APRDRG) mortality score [13].

Statistical Analysis

We compared the baseline characteristics of adults with CAP hospitalizations complicated with and without AKI-D. We utilized the chi-square test for categorical variables, the student’s t-test for normally distributed continuous variables, and the Wilcoxon rank-sum test for non-normally distributed continuous variables for descriptive analyses. We assessed changes in trends in AKI-D in CAP hospitalizations overall and in apriori defined demographic groups (age, sex, and race). We assessed changes in trend over time using the Cuzic trend test. We also performed a survey regression analysis to explore potential reasons for temporal changes in AKI-D overall by fitting a series of sequential models (Model 1: Only calendar year; Model 2: Model 1 + demographics; Model 3: Model 2 + acute/chronic comorbidities and procedures). We utilized survey logistic regression to evaluate the predictors of AKI-D and to estimate the impact of AKI-D on mortality and adverse discharge [8]. Finally, we calculated the trends of mortality over time and the attributable risk percent (ARP) of death, indicating the proportion of deaths that could potentially be avoided if AKI-D were eliminated. We constructed models ensuring no multi-co-linearity between covariates.

We performed all association and trend analyses using designated weight values by HCUP and considered a two-tailed p-value ≤ 0.01 as statistically significant. We utilized SAS 9.3 (SAS Institute Inc., Cary, North Carolina, US) for all analyses.

## Results

Trends of CAP hospitalizations complicated by AKI-D 

Between the years 2002 and 2013, there were 11,500,456 adult hospitalizations with CAP, out of which 36,975 (0.32%) had AKI-D.

Trends in demographic subgroups

**Figure 1:**
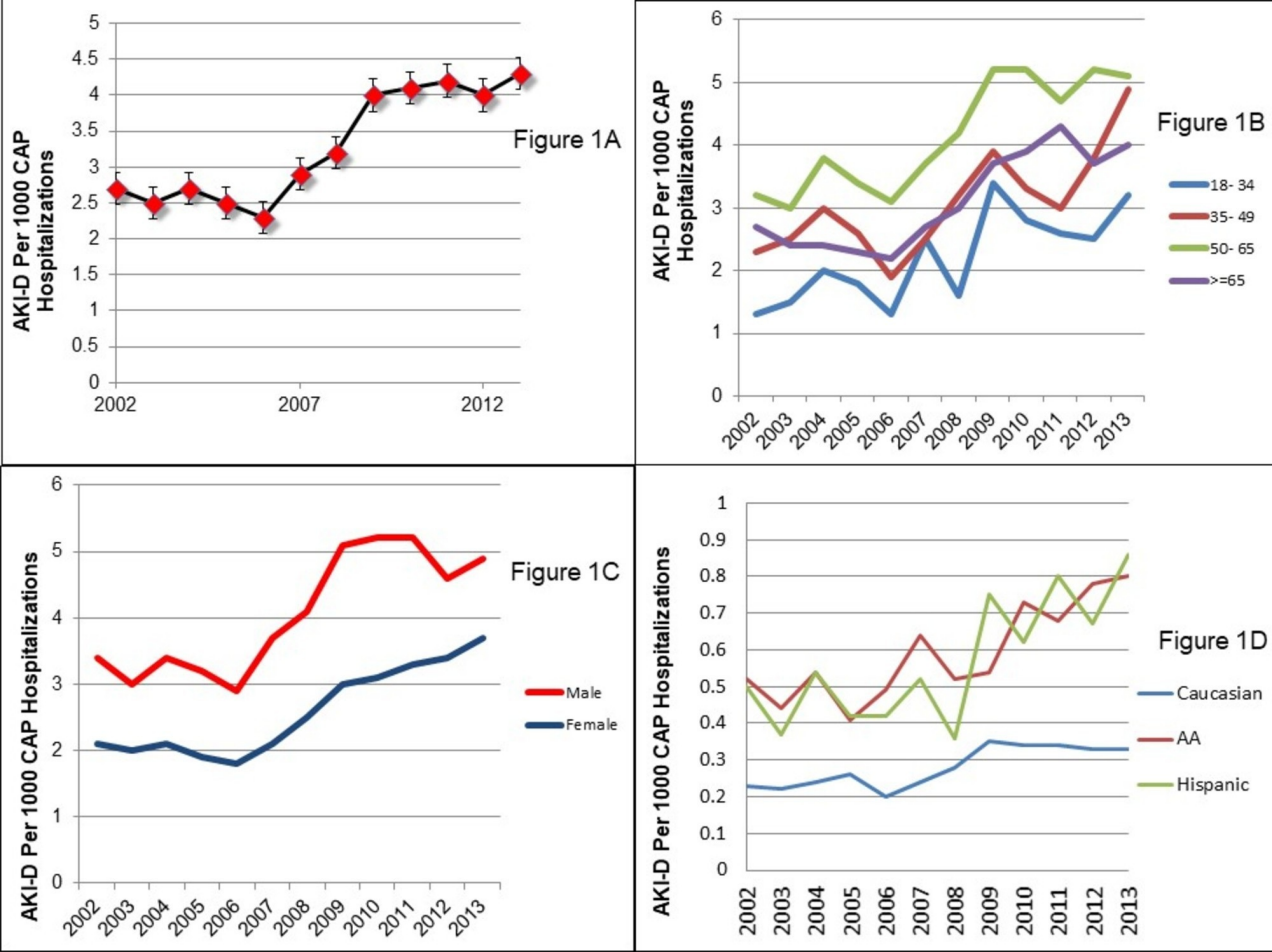
Trends in Acute Kidney Injury Needing Dialysis in Community-acquired Pneumonia Hospitalizations by (A) Overall; (B) Age; (C) Sex; and (D) Race.

There was a significant increase in AKI-D in CAP, from 2.7/1000 hospitalizations in 2002 to 4.3/1000 hospitalizations in 2013 (p<0.001) (Figure 1A). Patients between 18 and 34 years of age had the highest rate of increase in AKI-D (2.5 fold; 1.3/1000 hospitalizations in 2002 to 3.2/1000 hospitalizations in 2013). There was a decreasing rate of rise with increasing age (twofold in 35-49 years; 1.6 fold in 50-65 years, and 1.4 fold in ≥ 50 years) (Figure 1B). Although the proportion of AKI-D was higher in males, the rate of increase was higher in females (1.8 fold in females vs. 1.4 fold in males) (Figure 1C). By race, the rate of increase was similar between Hispanic/Latinos (1.8 fold) and African Americans (1.7 fold) with a lower rate in Caucasians (1.4 fold) (Figure 1D).

Explanation of trends in AKI-D

In a univariate analysis, CAP hospitalizations complicated by AKI-D increased by 6% per calendar year (OR 1.06; 95% CI 1.05-1.07). When adjusted for changes in demographics (age, sex, and race), the effect size was attenuated to 5% (aOR 1.05; 95% CI 1.04-1.06). After adjusting for changes in comorbidities and procedures, including mechanical ventilation and cardiac catheterization, this was further attenuated to 2% (aOR 1.02; 95% CI 1.01-1.03). Thus, adjusting for known demographic and comorbidity changes associated with AKI-D explained a substantial portion of the increasing trend, but there remained an unexplained increase of 2% per year (Table 1). 

**Table 1:** Sequential Models with Adjustment to Explain Trends of AKI-D in CAP Hospitalizations. ^a^Model 1: Unadjusted; ^b^Model 2: Adjusted for changes in age, sex and race; ^c^Model 3: Model 2 + Changes in comorbidities (HIV status, diabetes, hypertension, CKD, sepsis, heart failure, chronic liver diseases, liver cancer) and procedures (cardiac catheterizations and mechanical ventilation). CAP: community-acquired pneumonia; AKI-D: acute kidney injury requiring dialysis; HIV: human immunodeficiency virus; CKD: chronic kidney disease

	Unadjusted Odds Ratio/Year (95% CI)^a^	Adjusted Odds Ratio/Year 95% CI)^b^	Adjusted Odds Ratio/Year (95% CI)^c^
AKI-D in CAP Hospitalizations	1.061 (1.052 - 1.070)	1.052 (1.042 - 1.061)	1.029 (1.019 - 1.039)

Baseline characteristics of CAP hospitalizations with and without AKI-D

Patients with CAP hospitalizations complicated by AKI-D were younger (mean age 67.2 vs. 69 years; p<0.01), more likely to be male (58.1% vs. 46.6, p<0.01%), and had a higher proportion of the African American race (15.7% vs. 8.4%, p<0.01). They were more likely to be sicker (67.3% vs. 6.2% with APRDRF, p<0.01), more likely to have higher proportions of comorbidities, including diabetes mellitus, hypertension, chronic kidney disease (CKD), human immunodeficiency virus (HIV) status, liver disease, sepsis, heart failure, as well as procedures (cardiac catheterizations and mechanical ventilation) (Table 2). 

**Table 2:** Baseline Characteristics of CAP Hospitalizations with and without AKI-D. Both populations were compared utilizing the chi-square test, the Wilcoxon rank sum test, and survey regression depending on the distributions of individual variables. ¥ Quartile classification of the estimated median household income of residents in the patient's ZIP code. These values are derived from ZIP code-demographic data obtained from Claritas. CAP: community-acquired pneumonia; AKI-D: acute kidney injury requiring dialysis; APRDRG: All Patient Refined Diagnosis Related Group

Variable	CAP Hospitalizations Without AKI-D (n=11463481)	CAP Hospitalizations With AKI-D (n=36,975)	p
Age Mean (SE)	69.00 (0.08)	67.24 (0.21)	<0.01
18-34	4.78	3.29	
35-49	10.17	9.49	
50-64	19.85	25.59	
≥65	65.19	61.63	
Gender, (%)			<0.01
Male	46.64	58.08	
Female	53.33	41.92	
Race, (%)			<0.01
Caucasian	61.61	53.71	
African American	8.41	15.66	
Hispanic	5.68	10.16	
Others	3.72	5.25	
Missing	20.58	15.22	
APRDRG Mortality Scale, (%)			<0.01
1 & 2	72.16	4.5	
3	20.75	27.46	
4	6.16	67.3	
Concurrent Diagnosis, (%)			
Diabetes Mellitus	27.21	37.53	<0.01
Hypertension	51.43	66	<0.01
Chronic Kidney Disease	7.92	22.32	<0.01
Human Immunodeficiency Virus	1.06	1.85	<0.01
Acute or Chronic Liver Disease	3.5	12.43	<0.01
Sepsis	4.19	37.08	<0.01
Acute Myocardial Infarction	1.35	8.44	<0.01
Heart Failure	24.37	49.9	<0.01
Cardiac catheterizations	0.5	2.04	<0.01
Mechanical ventilation	3.59	49.05	<0.01
Zip code Income, (%) ¥			<0.01
0-25 percentile	27.55	28.38	
26-50 percentile	23.56	24.61	
51-75 percentile	20.15	19.9	
76-100 percentile	19.04	15.84	
Primary Payer, (%)			<0.01
Medicare/Medicaid	75.57	76.75	
Private	17.93	18.34	
Uninsured/Self pay	6.33	4.73	

Several patient and hospital demographics were statistically significant, however, they had small absolute differences.

Impact of AKI-D on outcomes

Unadjusted mortality was eight-fold higher in CAP hospitalizations with AKI-D vs. without AKI-D (32.4% vs. 4.4%) with a 10-fold increase in unadjusted odds of mortality (OR 10.65; 95% CI 10.02-11.31). After adjusting for confounders, while this was attenuated, AKI-D remained significantly associated with three-fold higher odds of mortality (aOR 3.08; 95% CI 2.88-3.30) (Table 3).

**Table 3:** Impact of AKI-D on In-hospital Mortality and Adverse Discharge in CAP Hospitalizations. Adjusted for changes in age, sex, race, hospital location, primary payer type, hospital bed size, hospital location, Zip code, income, APRDRG mortality scale. CAP: community-acquired pneumonia; AKI-D: acute kidney injury requiring dialysis; APRDRG: All Patient Refined Diagnosis Related Group

	Without AKI-D(%)	With AKI-D(%)	Unadjusted Odds Ratio (95% Confidence Interval)	Adjusted Odds Ratio (95% Confidence Interval)
Mortality	4.4	32.4	10.65 (10.02-11.31)	3.08 (2.88-3.30)
Adverse Discharge	37.4	44.6	2.43 (2.28-2.60)	2.09 (1.92 - 2.26)

**Figure 2:**
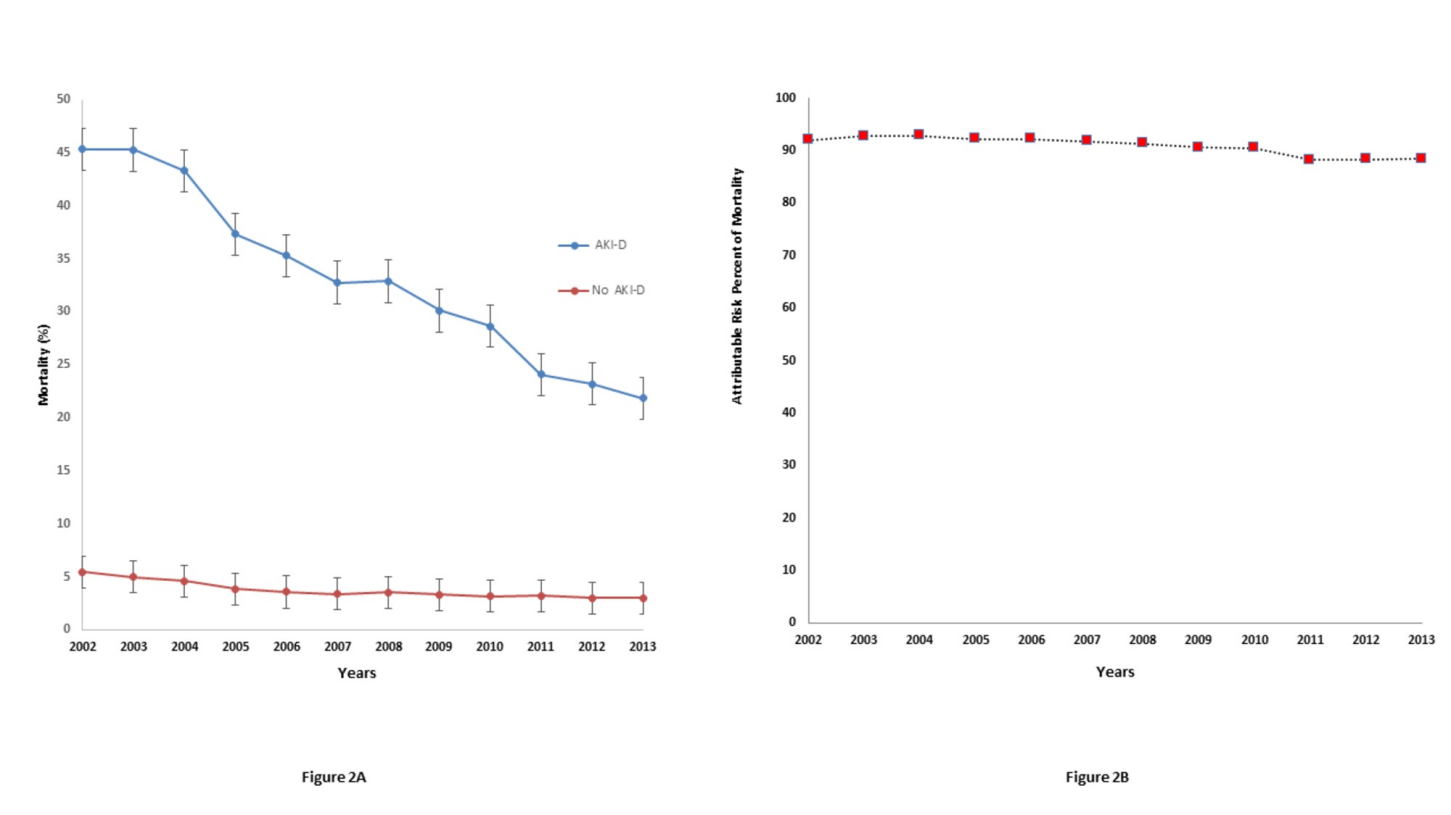
A. Trends of Mortality of CAP in AKI-D and No AKI-D Population (Bars represent standard errors.) B. Attributable Risk Percent of Mortality for AKI-D in CAP Hospitalizations. (Attributable risk percent of mortality was stable for AKI-D in CAP hospitalized patient from 2002 to 2013). AKI-D: acute kidney injury requiring dialysis

In-hospital mortality had decreased for both CAP hospitalizations with AKI-D (45.3% in 2002 to 21.8% in 2013) and without AKI-D (5.4% in 2002 to 3.0% in 2013) (Figure 2A). To evaluate the odds or mortality with increasing year, we compared the odds of mortality by consecutive year with the year 2002. The odds of mortality decreased every year from 1.01 (0.80–1.26) in 2003 to 0.33 (0.26–0.42) in 2013. This trend held true even after adjusting for patient demographics (age, sex, race), comorbidities (diabetes mellitus, hypertension, CKD, HIV, liver disease, sepsis, heart failure), and procedures (cardiac catheterization and mechanical ventilation) (Table 4). However, this was offset by an increase in the incidence of AKI-D, resulting in a stable attributable risk percent from 2002-2013 (Figure 2B).

**Table 4:** Adjusted Odds Ratios for In-hospital Mortality for AKI-D in CAP Hospitalizations. Adjusted for demographics, acute/chronic comorbidities, procedures, and hospital-level characteristics. CAP: community-acquired pneumonia; AKI-D: acute kidney injury requiring dialysis

Year	Unadjusted OR	Adjusted OR
	95 CI%	95 CI%
2002	Referent	
2003	1.01 (0.80 - 1.26)	0.96 (0.70 - 1.32)
2004	0.94 (0.74 - 1.18)	0.78 (0.56 - 1.07)
2005	0.72 (0.57 - 0.92)	0.82 (0.60 - 1.14)
2006	0.66 (0.51 - 0.86)	0.68 (0.48 - 0.95)
2007	0.58 (0.45 - 0.75)	0.65 (0.46 - 0.92)
2008	0.59 (0.46 - 0.75)	0.49 (0.36 - 0.67)
2009	0.51 (0.40 - 0.64)	0.58 (0.43 - 0.79)
2010	0.49 (0.39 - 0.61)	0.55 (0.41 - 0.74)
2011	0.38 (0.30 - 0.49)	0.42 (0.31 - 0.57)
2012	0.37 (0.29 - 0.48)	0.47 (0.35 - 0.65)
2013	0.33 (0.26 - 0.42)	0.45 (0.33 - 0.62)

**Figure 3:**
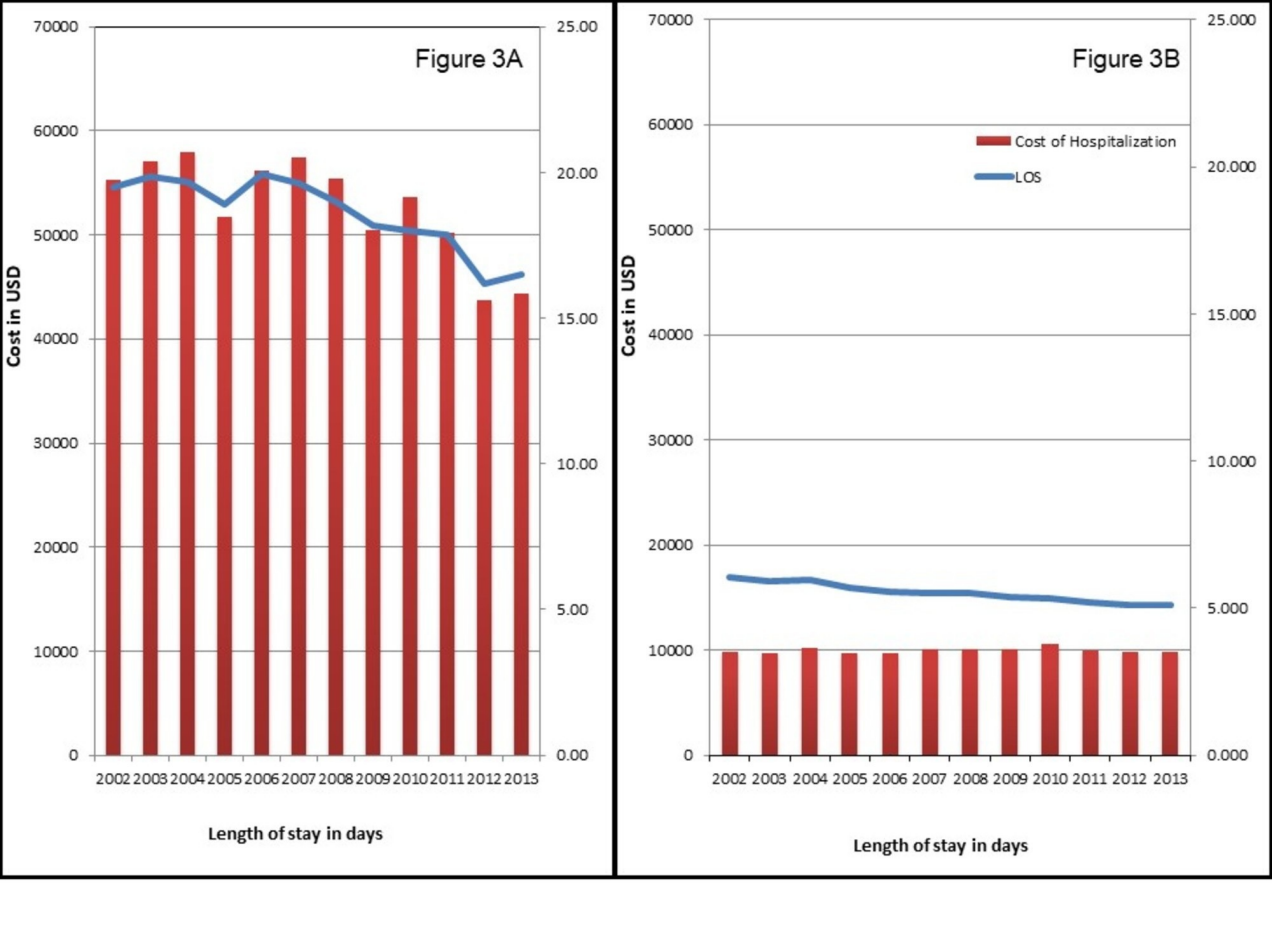
Trends in Length of Stay and Cost for CAP Hospitalizations with AKI-D (A) and without AKI-D (B). AKI-D: acute kidney injury requiring dialysis

Similarly, hospitalizations with AKI-D had higher proportions of adverse discharge (44.6% vs. 37.4%), with a two-fold higher adjusted odds of adverse discharge (aOR 2.09; 95% CI 1.92-2.26) (Table 2). AKI-D was associated with a four-fold higher LoS (18.6 days vs. 5.5 days, p<0.01), and fivefold higher cost (USD 52,785 vs. 9,888, p<0.01). The LoS and inflation-adjusted cost of hospitalization for CAP hospitalizations stayed stable from 2002-2013 regardless of AKI-D status, however, both LoS and cost remained higher for AKI-D for all years (Figures 3A-3B).

Significant predictors of AKI-D in CAP hospitalizations

**Figure 4:**
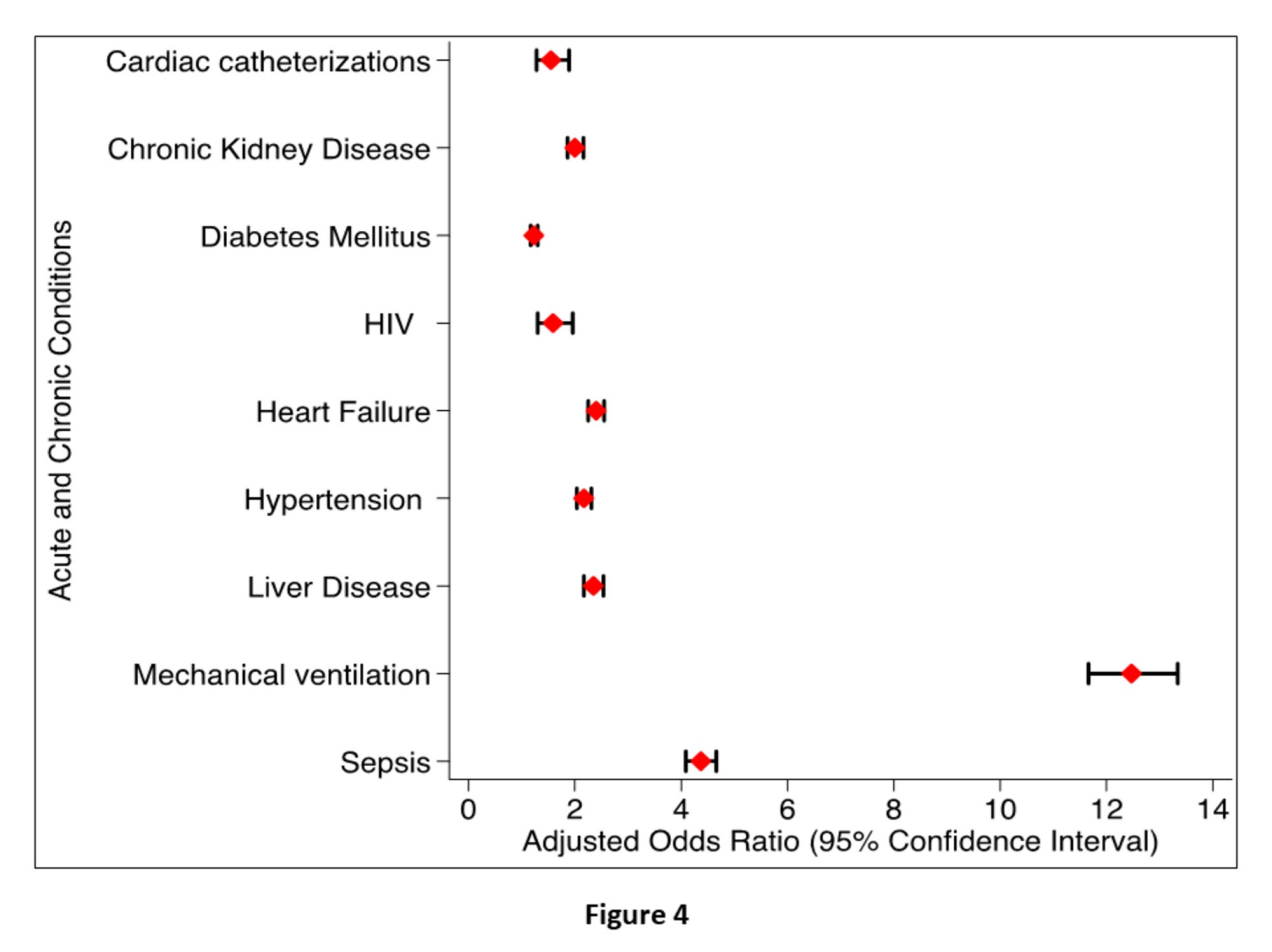
Acute and Chronic Comorbidities and Procedures Associated with AKI-D in CAP Hospitalizations. The strongest association of AKI-D was requiring mechanical ventilation during hospitalization. AKI-D: acute kidney injury requiring dialysis; CAP: community-acquired pneumonia

Figure 4 shows the significant associations of AKI-D in CAP hospitalizations.

The covariate with the highest adjusted odds ratio for AKI-D was requiring mechanical ventilation during hospitalization (aOR 12.47; 95% CI 11.66-13.34). In addition, sepsis (aOR 4.37; 95% CI 4.09-4.66), heart failure (aOR 2.40; 95% CI 2.25-2.55), and CKD (aOR 2.00; 95% CI 1.86-2.16) were also independent predictors.

## Discussion

Utilizing a large, nationally representative database, we demonstrated that the proportion of CAP hospitalizations complicated by AKI-D has increased over the last decade. This increase is different in multiple demographic subgroups. We also showed that while changes in demographics and comorbidities explain a substantial proportion of this increase, it does not completely account for it. Finally, we show that AKI-D is associated with significantly worse in-hospital outcomes in CAP hospitalizations and while the adjusted mortality has decreased over time, this is offset by an increased incidence, leading to stable attributable mortality.

Previous studies considering AKI in CAP hospitalizations have been limited to a single center and/or year [7-8]. An analysis from the Genetic and Inflammatory Markers of Sepsis study cohort found that AKI was common in CAP and in those patients even with mild CAP or having an uncomplicated hospital course, AKI still developed in one-quarter of the participants and was associated with significant morbidity and mortality [8]. However, this study was single-center, did not specifically consider the dialysis requirement in AKI, and could not elucidate national trends in AKI-D. We significantly added to the literature on this complication in CAP hospitalizations by using a nationally representative database.

We demonstrated that AKI-D has inexorably increased over the study period and changes in demographic factors and comorbidities cannot completely account for its rise. Whether the rise in AKI-D is linked to the increased use of nephrotoxins, such as contrast studies, or the decreased in-hospital mortality of CAP patients, thereby having more patients surviving and requiring renal replacement therapy is currently unclear. However, given the small absolute change of 2% in in-hospital mortality from 2002–2013, we suspect that this likely plays a smaller role. Notably, a sharp increase occurred from 2006–2009. This finding was seen across all subgroups for age, gender, and race. It is currently unclear why there was a large increase in AKI-D during these years, however, this may be related to changes in the coding of these conditions or changes in the NIS database. This finding warrants further evaluation using a more granular database to see if this holds true in other samples. A recent study from the NIS database of the general population showed that the temporal trend in six diagnoses: sepsis, hypertension, respiratory failure, hemorrhagic disorders, shock, and liver disease, completely accounted for the temporal rise of AKI-D in the general population [14]. Although, accounting for these comorbidities explained a substantial proportion of the rise in CAP hospitalizations, one-third of the temporal rise could not be accounted for. This highlights that AKI-D in specific disease conditions may be different from that in the general population and underscores the need for studies using large-scale, granular, patient-level data to delineate risk factors in specific patient populations and disease conditions.

In addition, we demonstrate that AKI-D has been rising at different rates in several demographic subgroups. Although older age has been known to be significantly associated with AKI-D and had an overall higher proportion of AKI-D [15], we show that the youngest age group in our analysis was associated with a higher rate of temporal increase in AKI-D. This is an important finding since there has been an increasing focus on AKI in elderly individuals [16], however, our analysis also identifies an increasing temporal rate of AKI-D in younger individuals, which have previously been considered “low risk.” This highlights the importance of the early recognition and prevention of AKI in young individuals before they progress to needing dialysis. We also demonstrate that the rate of AKI-D temporal rise was higher in women although the absolute proportion was higher in men. It has been shown before that the risk of AKI is higher in men as compared to women, although temporal trends in women have not been explored and our study is, to the best of our knowledge, the first to assess temporal trends by gender. Finally, we demonstrate that African Americans and Hispanic/Latinos have the highest rates and absolute proportion of AKI-D. This has been shown before in other populations and whether it is linked to ethnicity-specific genetic polymorphisms or socioeconomic factors is still a matter of debate [17-18].

We also identified multiple significant predictors of AKI-D in CAP hospitalizations. The strongest predictor was requiring mechanical ventilation, which is likely a surrogate for the significant severity of CAP and the overall illness of the patient, predisposing to both the development and worsening of AKI [19]. Another strong predictor, sepsis, has been linked to AKI-D in a variety of scenarios and represents worsening CAP, leading to sepsis and end-organ damage [5]. Although other comorbidities are also independently associated with AKI-D, CKD was one of the strongest predictors. Since AKI and CKD are likely interconnected, this shows the importance of using baseline kidney function for the risk stratification of the development of AKI [20-21]. However, these predictors need to be accurately quantified using patient-level data. Together with biomarkers like serum creatinine and blood urea nitrogen levels, these predictors could represent a preliminary step towards the creation of a recursive score useful in risk-stratifying CAP hospitalizations for AKI-D. Identification of AKI continues to rely on creatinine, blood urea nitrogen (BUN), and urine output, which often do not increase until hours to days after the injury. Novel biomarkers, such as neutrophil gelatinase-associated lipocalin (NGAL) and kidney injury molecule 1 (KIM-1), while not having had great success at predicting the development of AKI are associated with the increased odds of progression of AKI after cardiac surgery [22]. While this dataset does not capture urinary biomarker levels, it would be of interest to see how a risk score that combined clinical predictors and urinary predictors would perform in CAP patients.

Finally, we demonstrated that AKI-D is significantly and independently associated with in-hospital mortality after accounting for several potential confounders. Interestingly, the adjusted mortality has decreased every year, which is likely due to better in-hospital care, as adjusting for patient demographics, patient comorbidities, and procedures did not substantially affect this trend. Despite decreasing mortality, due to the increasing incidence of AKI-D in CAP hospitalizations, the attributable risk of mortality and thus its burden has remained stable. AKI-D is associated with both longer LoS and cost. AKI-D was associated with an excess cost of USD 42,000 per patient, leading to a total cost of 1.5 billion attributable to AKI-D over the last decade in CAP hospitalizations. AKI-D is also strongly associated with an adverse discharge, which might be due to the fact that AKI is linked strongly to the future development of ESRD. Therefore, these patients with AKI-D might need specialized, nursing home care post discharge [20]. This demonstrates the burden of AKI-D in survivors of CAP hospitalization.

There are limitations to our analysis. Instead of laboratory data, we rely on administrative diagnosis codes for defining AKI-D, which may incorrectly identify patients who were initiated on dialysis for other reasons, such as for metabolic control and fluid management. However, we suspect this number to be small and AKI-D codes have been demonstrated to have >90% validity. As this is an administrative database, we did not have access to important clinical factors, such as laboratory data, timing of dialysis initiation, or dialysis-related complications, which are all important factors that affect morbidity and mortality. With a lack of follow-up information, we cannot define outcomes in AKI-D survivors. We are unable to account for unmeasured confounders that could be associated with increased AKI-D in CAP patients although we attempted to address severity and mortality risk using APRDRG scores – a prevalent standard in adjusting for disease burden in administrative databases. We cannot account for patients who had repeated hospitalizations for CAP and AKI-D since we used hospitalization-level not patient-level data. Despite these limitations, the benefit of using a large, multi-center, representative sample preserves the overall validity of associations reported.

## Conclusions

Currently, there are no effective therapies for AKI and care is only supportive. In this scenario, the cornerstone for AKI management is early diagnosis, risk stratification, and the prevention of AKI by avoiding nephrotoxins (e.g. aminoglycosides, non-steroidal anti-inflammatory drugs (NSAIDs), parenteral contrast media (CM)), and volume depletion, etc. By demonstrating that severe AKI in CAP hospitalizations is on the rise, we hope that resources will focus on this problem, both from research and clinical practice perspectives, in order to both prevent and improve the outcomes of this devastating complication.
